# OsRRM, an RNA-Binding Protein, Modulates Sugar Transport in Rice (*Oryza sativa* L.)

**DOI:** 10.3389/fpls.2020.605276

**Published:** 2020-12-08

**Authors:** Derui Liu, Lina Xu, Wei Wang, Shuwen Jia, Sukui Jin, Jiping Gao

**Affiliations:** ^1^Jiangsu Co-Innovation Center for Modern Production Technology of Grain Crops, Key Laboratory of Crop Genomics and Molecular Breeding of Jiangsu Province, Key Laboratory of Crop Genetics and Physiology of Jiangsu Province, College of Agriculture, Yangzhou University, Yangzhou, China; ^2^National Key Laboratory of Plant Molecular Genetics, CAS Center for Excellence in Molecular Plant Sciences, Shanghai Institute of Plant Physiology and Ecology, Chinese Academy of Sciences, Shanghai, China; ^3^Innovation Academy for Seed Design, Chinese Academy of Sciences, Beijing, China

**Keywords:** RNA binding protein, *Spen*-like, *Oryza sativa*, sugar transport, sugar signaling

## Abstract

Sugar allocation between vegetative and reproductive tissues is vital to plant development, and sugar transporters play fundamental roles in this process. Although several transcription factors have been identified that control their transcription levels, the way in which the expression of sugar transporter genes is controlled at the posttranscriptional level is unknown. In this study, we showed that OsRRM, an RNA-binding protein, modulates sugar allocation in tissues on the source-to-sink route. The *OsRRM* expression pattern partly resembles that of several sugar transporter and transcription factor genes that specifically affect sugar transporter gene expression. The messenger RNA levels of almost all of the sugar transporter genes are severely reduced in the *osrrm* mutant, and this alters sugar metabolism and sugar signaling, which further affects plant height, flowering time, seed size, and starch synthesis. We further showed that OsRRM binds directly to messenger RNAs encoded by sugar transporter genes and thus may stabilize their transcripts. Therefore, we have uncovered the physiological function of OsRRM, which sheds new light on sugar metabolism and sugar signaling.

## Highlights

- Sugar allocation is important in plant growth, development, and crop yield.
- Sugar transporter genes are under control of a novel RNA-binding protein, OsRRM.
- OsRRM binds to sugar transporter gene transcripts and may act to stabilize the mRNA.
- Our research results will lead to a new direction in the field of crop improvement.

## Introduction

Carbon assimilation occurs during photosynthesis. Excess assimilated carbon is stored in the form of starch either in leaves, as has been shown in *Arabidopsis* (Zeeman and Ap Rees, 1999), or in the sink tissues after conversion from soluble sugars, such as sucrose, that are transported from the photosynthetic tissues in cereals (Nakano et al., 1995). Sucrose can be hydrolyzed into the monosaccharides glucose and fructose. These three types of sugar are widely distributed to heterotrophic sink tissues (Rees, 1994).

Sugars have multiple functions in all organisms, including their well-known function as energy resources. In plants, sugars are needed for cell wall biosynthesis (Carpita, 1996) and for the synthesis of other carbohydrate polymers, such as starch (Hanes, 1940). In addition, sugars can be used as precursors for the biosynthesis of a diverse range of metabolic intermediates. Sugars are also related to pathogen susceptibility (Yamada et al., 2016). Thus, maintaining the homeostasis of sugar content and allocation is vital to plant growth and development (Riesmeier et al., 1993, 1994; Eveland and Jackson, 2012; Lastdrager et al., 2014).

Sugar preservation during sugar translocation to sink tissues is necessary for plant growth and reproduction (Patrick and Offler, 1996), for which plants have evolved various sugar transport systems. Sugar transport between adjacent cells can occur *via* sugar transporters at the plasma membrane (Williams et al., 2000) or through plasmodesmata (Turgeon and Medville, 1998). Within cells, sugar transport takes place through the chloroplast envelope membrane (Weber et al., 2000) and the vacuolar membrane (Wormit et al., 2006). For long distance transport, sugars are first loaded into phloem sieve cells where they are further transported to sink tissues (Ruiz-Medrano et al., 2001; Lalonde et al., 2004).

In the context of controlling sugar translocation, attention has been focused on isolating and characterizing sugar transporter genes. For example, *Oryza sativa* sucrose transporter 1 (OsSUT1), a protein that localizes to the plasma membrane, retrieves sucrose from the apoplasm and transports it into the phloem sieve cells where it is further transported to the filling grains (Scofield et al., 2007). The tonoplast monosaccharide transporters TMT1 and TMT2 transport monosaccharides driven by proton gradients in *Arabidopsis* (Wormit et al., 2006). Sugars will eventually be exported, and transporters 11 and 12 (SWEET11 and 12) were identified as sucrose efflux transporters in *Arabidopsis* that are important for phloem transport (Chen et al., 2012); SWEET9 plays a fundamental role in nectar production (Lin et al., 2014), and the expression of *SWEET11* and *15* is necessary for apoplasmic seed filling in rice (Ma et al., 2017; Yang et al., 2018). The plastidic sugar transporter (pSuT) in *Arabidopsis* influences both the vegetative-to-reproductive growth transition and the architecture of the inflorescence (Patzke et al., 2019), and flowering time control genes may act upstream of sugar transporter genes; this is exemplified by the observation that *FLOWERING LOCUS T* (*FT*), a central flowering integrator, controls *SWEET10* expression in *Arabidopsis* (Andrés et al., 2020). In addition, overexpression of *Arabidopsis* phloem-specific sucrose transporter 2 (*AtSUC2*) in rice increased yield (Wang et al., 2015), and overexpression of rice monosaccharide transporter 6 (*OsMST6*) (Wang et al., 2008) in *Arabidopsis* enhanced plant tolerance to abiotic stresses (Monfared et al., 2020).

It has been shown that the expression of these sugar transporter genes is under transcriptional and posttranscriptional control (Kühn and Grof, 2010; Xu et al., 2018). Low concentrations of sugars inhibit the transcription of several sugar transporter protein (*STP*) genes in *Arabidopsis* (Cordoba et al., 2015). However, there are very few reports describing factors that specifically regulate the expression of these genes, except for two transcription factors, *O. sativa* DNA-binding with one finger 11 (OsDOF11) and aleurone-specific NF-YB1, which coordinately control the expression of *SUT1*, *OsSWEET11*, and *OsSWEET14* (Wu et al., 2018), or trigger the expression of *SUT1*, *SUT3*, and *SUT4*, thus regulating grain filling (Bai et al., 2016), respectively.

RNA-binding proteins (RBPs), as master regulators, govern all aspects of gene regulation at the transcriptional and posttranscriptional levels (Glisovic et al., 2008; Hogan et al., 2008; Lorkovic, 2009). The functions of RBPs in animal cells have been well studied, but less effort has been directed toward isolating RBPs from species in the plant kingdom. We previously reported a Split ends (Spen)-like family of genes that are thought to encode RBPs. There are three *Spen*-like genes in the rice genome, two of which, *OsRRMh* and *OsRRM*, have been cloned. *OsRRMh* was reported to be an important governor of flowering time control (Liu and Cai, 2013). *OsRRM* shows the highest sequence similarity to *OsRRMh*, and the structure of the *OsRRM* gene was first described by Chen et al. (2007). The predicted protein consists of two RNA recognition motifs (RRMs), one Spen paralog and an ortholog C-terminal (SPOC) domain and a Leucine-zipper (L-ZIP) motif. Because many RBPs contain RRMs, and this RNA-binding motif is the best characterized one in nearly all organelles (Kenan et al., 1991; Dreyfuss et al., 1993; Burd and Dreyfuss, 1994), the OsRRM protein was proposed to be an RBP. However, its function as an RBP has never been studied yet except for the observation of retarded growth that occurs when the gene is ectopically overexpressed (Chen et al., 2007).

In this study, we examined the effects of the *osrrm* null-mutation on rice plant growth and development and sugar metabolism. The *osrrm* mutant exhibits dwarfing, retarded flowering, smaller seeds, changes in amylose and amylopectin synthesis, and impaired sugar transport. Based on the changes in the expression of starch synthesis-related genes and sugar transporter genes, we believe that the retarded growth of *osrrm* mutant plants results from aberrant sugar transport. We further show that OsRRM directly binds to transcripts of sugar transporter genes and thus may act to stabilize them. In summary, OsRRM may confer its functions on sugar metabolism and sugar signaling to control rice growth and development by modulating the expression of sugar transporter genes.

## Materials and Methods

### Plant Materials and Growth Conditions

The *japonica* rice (*O. sativa* L.) cultivar “Hwayoung” (WT) and the *osrrm* mutant (PFG_2D-01214) were acquired from Pohang University of Science and Technology, South Korea (Jeong et al., 2002). The rice cultivar “Zhonghua 11” (ZH11) was used for sugar and abscisic acid (ABA) treatment assays. Rice plants were grown either under natural environmental conditions in the Song Jiang experimental field (31.0322° N, 121.2277° E) at the Shanghai Institute of Plant Physiology and Ecology for phenotypic analysis of plant architecture, seed size, and seed weight or in the greenhouse at 28°C (day) and 22°C (night) under natural daylight (383 μmol m^–2^ s^–1^) for other analyses. Photoperiods were 10 h (light)/14 h (dark) for the short-day condition and 14 h (light)/10 h (dark) for the long-day condition in the greenhouse. For plant treatment with exogenous sugars, seeds were germinated and grown in H_2_O for 5 days, followed by immersing the roots in H_2_O with exogenous sugar added, and the seedlings were allowed to grow in the dark overnight.

### *OsRRM* Mutant Complementation

The *OsRRM* promoter region, a 2-kb fragment upstream of the start codon of *OsRRM* (Os09g12730), and the *OsRRM* coding region (3,018 bp) were amplified either from ZH11 genomic DNA or from the cDNA clone J023129A05^1^ using the gene-specific primers shown in Supplementary Table 1 and KOD PLUS DNA polymerase (Toyobo). The PCR-amplified fragments were inserted tandemly into the binary vector pCAMBIA2301, and were verified by Sanger sequencing. *Agrobacterium tumefaciens* strain EHA105 harboring the complementation construct was used to transform immature embryos of the *osrrm* mutant following the protocol described by Liu et al. (1998), and transformants were screened on G418-containing medium.

### Southern Blot Analysis

Total DNA (20 μg), isolated from leaves, was digested with restriction enzymes, separated on a 0.7% (w/v) agarose gel, and blotted onto a nylon membrane (Amersham) and DNA detection was performed using an enhanced chemiluminescence (ECL) nucleic acid labeling and detection kit (Amersham). The probe used was an 884-bp fragment of the hygromycin resistance gene that was PCR-amplified with primers *Hyg*-F and *Hyg*-R (Supplementary Table 1).

### Semiquantitative Real-Time PCR and Quantitative Real-Time PCR Analysis

Total RNA extracted from freshly harvested tissues with the RNAprep pure Plant Kit (Tiangen) was digested with RNase-free DNase (Takara) to remove contaminating genomic DNA. First-strand cDNA was synthesized from total RNA (1 μg) using ImProm-II^TM^ reverse transcriptase (Promega).

Semiquantitative real-time (RT)-PCR was carried out using Taq MasterMix (*CW*-BIO). The rice *Actin* gene was used as the internal control to normalize gene expression. Quantitative real-time PCR (qRT-PCR) was performed using SYBR Green II detection (Bio-Rad). Rice *UBQ10* was used as the internal control for normalization of gene expression. The rice flowering control-related genes were amplified as described by Liu and Cai (2013). The sugar transport-related genes were amplified as previously described (Cho et al., 2010; Hirose et al., 2010; Zhang et al., 2010; Eom et al., 2011). The primers used are shown in Supplementary Table 1.

### Scanning Electron Microscopy Observation of Glumes

Dry seeds were pretreated as described by Yang et al. (2019) before scanning electron microscopy observation (S-3000N; Hitachi). Cell number counting was performed using ImageJ software.

### Western Blot Analysis

Western blotting was performed according to Zhang et al. (2006). Protein samples (∼20 μg) were separated on 8 or 10% sodium dodecyl sulfate-polyacrylamide gel electrophoresis (SDS-PAGE) gels, after which they were transferred to a polyvinylidene fluoride (PVDF) membrane (GE Healthcare). The ECL Plus Western Blotting Detection Kit (GE Healthcare) was used for detection.

The antibodies used were: (1) anti-AGPase small subunit antibody (1:1,000; a gift from Michael J. Emes, University of Guelph), (2) anti-GBSS1 antibody (1:5,000; custom-made by Immunogen), (3) anti-SSI antibody (1:5,000), (4) anti-SSIIa antibody (1:1,000), (5) anti-SBEI antibody (1:5,000), (6) anti-SBEIIb antibody (1:5,000), (7) anti-ISA1 antibody (1:1,000), and (8) anti-SP antibody (1:5,000) (#s 3–8 were custom-made by *CW*-BIO; #s 1–8 are rabbit polyclonal antibodies), (9) anti-ACTIN mouse monoclonal antibody (1:10,000; Proteintech^TM^), (10) anti-His mouse monoclonal antibody (1:2,000; Abmart), and (11) horseradish peroxidase-linked goat anti-rabbit or anti-mouse secondary antibodies (1:10,000; *CW*-BIO).

### Grain Quality Measurement

The endosperms from the deglumed dry rice grains were ground to powder. The apparent amylose content (AAC) and total starch content were measured using the iodine colorimetry assay as described by Williams et al. (1970) and the total starch assay kit (K-TSTA) (Megazyme), respectively. The total soluble sugar content was determined using the anthrone method (Wang et al., 2013). To measure the chain length distributions of amylopectin, endosperm powder was digested with isoamylase (Sigma-Aldrich) before mounting on and analysis using high-performance anion-exchange chromatography with pulsed amperometric detection (HPAEC-PAD) (Wang et al., 2013). The absolute percentage changes for each degree of polymerization (DP) are shown in line charts.

### Measurement of Soluble Sugar Content

Freshly ground plant samples (100 mg) were mixed with 1 ml deionized H_2_O containing 0.02% (w/v) sodium azide, the solutions were filtered through a 0.2-μm microfuge spin filter and were analyzed by high-performance liquid chromatography (HPLC). Concentrations of glucose, fructose, and sucrose were calculated using the individual standards (Klann et al., 1993).

### Sugar Export Activity Assay

Phloem exudate liquid was collected following the protocol described by Eom et al. (2011). The top leaf blades of 4-week-old plants were cut at the bottom at the end of the day followed by soaking in 500 μl 15 mM ethylenediamine tetraacetic acid (EDTA) solution (pH 7.25) for 16 h in the dark. The exudate solutions were filtered through a 0.2-μm microfuge spin filter followed by HPLC analysis of the concentrations of glucose, fructose, and sucrose. The concentration of C6 sugar per gram fresh weight (FW) of sample was calculated and defined as sugar export activity.

### Construction of the *pOsRRM*::*OsRRM* gDNA-GUS Plasmid and Histochemical GUS Staining

For the *pOsRRM*::*OsRRM* gDNA-β-Glucuronidase gene (GUS) construct, the *pOsRRM*-*OsRRM* genomic fragment was amplified from ZH11 genomic DNA using the *OsRRMp*-5’/*OsRRM*-3’ primer pair shown in Supplementary Table 1. The amplified DNA fragment was inserted upstream of the *GUS* gene between the *Bam*HI and *Pst*I sites in the p1300GN-GUS vector using the In-fusion enzyme (Clontech) and were verified by Sanger sequencing.

GUS staining was performed using the protocol described by Edwards et al. (1990) with the following changes: The stained detached tissues were either decolored with 70% ethanol and directly photographed with a stereoscopic microscope (BX51 plus DP70; Olympus) or fixed overnight in FAA solution [50% ethanol:formaldehyde:acetic acid, 90:5:5 (v/v/v)], followed by dehydration in a graded ethanol series, vitrification by dimethybenzene, and embedding in paraffin. Thin sections (8 μm) were cut and dewaxed with dimethylbenzene and rehydrated in a graded ethanol series. The sections were photographed using a stereoscopic microscope (DP72; Olympus).

### Expression and Purification of Recombinant Truncated OsRRM

A 735-bp fragment of the OsRRM CDS from the start codon to the second RRM was amplified with KOD PLUS DNA polymerase (Toyobo). The fragment was inserted into the *Sal*I site in pET32b, downstream of the T7 promoter and in-frame with Trx- and His-tag(s). Plasmids pET32b-OsRRM (1-735) and pET32b were transformed into *Escherichia coli* strain Rosetta (DE3). Fusion protein production was induced at 37°C for 3 h in Luria-Bertani (LB) medium supplemented with 0.5 mM isopropyl β-D-1-thiogalactopyranoside (IPTG). The fusion proteins were purified on Ni-NTA columns under native conditions (Qiagen).

### RNA Electrophoretic Mobility Shift Assay

The RNA electrophoretic mobility shift assays (REMSAs) with total RNA were performed according to Ambrosone et al. (2015) with the following changes: total RNA was isolated from stems and leaf sheaths of ZH11 plants before heading using the RNAprep pure Plant Kit (Tiangen); total RNA was labeled using the RNA 3′ End Biotinylation Kit (Thermo Fisher Scientific); the labeled RNA was incubated with 7.5 μg pET32b or pET32b-OsRRM (1-735) for 30 min; the LightShift^®^ Chemiluminescent RNA EMSA Kit (Thermo Fisher) was used; the binding reaction was detected with the Nucleic Acid Detection Module Kit (Thermo Fisher).

For REMSAs with specific messenger RNAs (mRNAs), the full-length *OsSUT2*, *OsTMT1*, *OsTMT2*, and *SBEIIb* mRNAs were amplified using the primer pairs shown in Supplementary Table 1, then subcloned into the pSK vector. The *in vitro* transcriptions were performed using the method described by Dong et al. (2005) except that UTP was used to replace Dig-UTP, and the Transcript Aid T7 High Yield Transcription Kit (Thermo Scientific) was used instead of the Roche transcription system. REMSAs were performed using the LightShift^®^ Chemiluminescent RNA EMSA Kit (Thermo Fisher Scientific).

### Statistical Analysis

Statistical analyses were calculated in Excel with built-in formulas. The *p-*values were calculated using two-tailed Student’s unpaired *t*-test analysis for binary comparison or with one-way ANOVA and Tukey’s *post hoc* honest significance test for comparisons of more than two genotypes.

## Results

### The *osrrm* Mutation Causes Dwarfing, Late Flowering, and Smaller Seed Size

To characterize the biological functions of OsRRM, a T-DNA insertion *osrrm* mutant (ID: PFG_2D-01214) was acquired from the Rice T-DNA insertion sequence database (RISD) collection. The T-DNA insertion site in *osrrm* was confirmed by PCR using gene- and T-DNA-specific primers (Supplementary Figure 1A). The homozygous mutant was further isolated from segregating progeny (Supplementary Figure 1B). Southern blot analysis confirmed that *osrrm* carries only one T-DNA insertion (Supplementary Figure 1C). Semiquantitative RT-PCR analysis indicated that transcripts of the endogenous *OsRRM* gene were absent in the isolated homozygous mutant (Supplementary Figure 1D); therefore, the *osrrm* allele is a null mutant.

**FIGURE 1:**
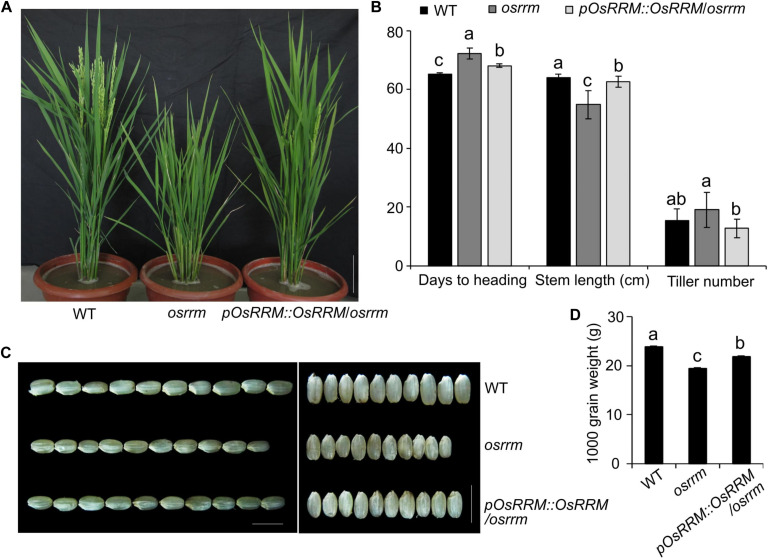
Characterization of plant architecture and seed morphology in the *osrrm* mutant and the *pOsRRM::OsRRM/osrrm* complemented transgenic line. **(A)** Plant architecture of wild-type (WT), *osrrm*, and *pOsRRM::OsRRM/osrrm* plants at the heading stage. Bar = 10 cm. **(B)** Days to heading, stem length, and tiller number in the WT, *osrrm*, and *pOsRRM::OsRRM/osrrm* plants, *n* = 20. **(C)** Grain size in WT, *osrrm*, and *pOsRRM::OsRRM/osrrm* plants. Bar = 5 mm. **(D)** Grain weight analysis. 1,000 grains were weighed for each line. The different letters in panels **(B,D)** indicate statistically significant differences analyzed with one-way ANOVA followed by Tukey’s test (*p* < 0.05). The measurements were repeated twice with the plants grown and seeds harvested in the two experimental years with similar results. For all variables with the same letter or overlaps with the same letter (such as “a” and “ab”), the difference between the means is not statistically significant. In contrast, if two variables have different letters, they are significantly different.

The *osrrm* mutant displayed dwarfing and late-flowering phenotypes (Figure 1A). The flowering time and days to heading were delayed in the *osrrm* plants (72 days) compared with wild type (WT) (65 days). The delayed flowering was accompanied by a reduction in the stem length from 64 to 55 cm and an increase in the tiller number from 15 to 19 (Figure 1B).

The dwarf and late-flowering phenotypes may indicate abnormalities in assimilation, which result in smaller seeds (Figure 1C) and reduced seed weight (Figure 1D). The dehulled grain weight of the *osrrm* mutant was ∼90% that of WT (Figure 1D). Because cell numbers and cell sizes in the palea and lemma determine seed size in cereals (Song et al., 2007), we examined *osrrm* and WT seeds using scanning electron microscopy (SEM). The result showed that lemma cells in the *osrrm* mutant are smaller than those in WT; the cell number in one visual field was 7 for WT and 9 for the *osrrm* mutant (Supplementary Figure 2A), but the total number of cells along the longitudinal axis of the glume is the same in both WT and the *osrrm* mutant (Supplementary Figure 2B).

The phenotype of the *osrrm* mutant could be complemented by introducing a WT copy of the *OsRRM* coding sequence (CDS) driven by the native *OsRRM* promoter (*pOsRRM*). Two homozygous transgenic lines were obtained (Supplementary Figure 3), and line-1, named *pOsRRM::OsRRM*/*osrrm*, was used for subsequent assays (Figure 1). *OsRRM* expression was restored to the normal level in the *pOsRRM::OsRRM/osrrm* transgenic plants (Supplementary Figure 3B).

**FIGURE 2:**
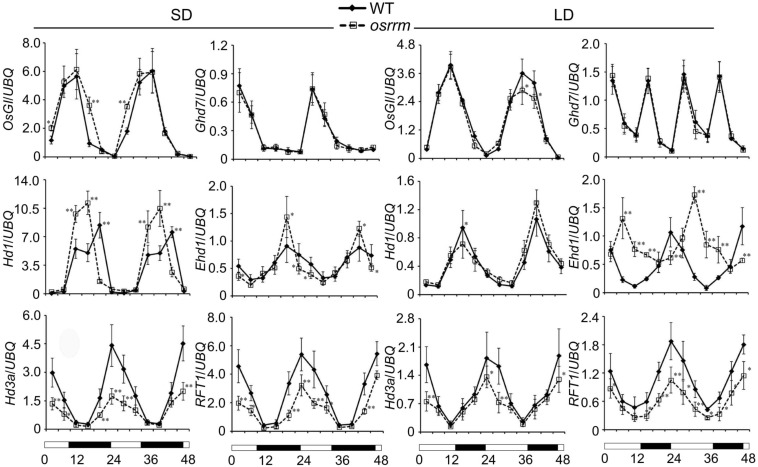
Circadian rhythms of flowering control gene expression in wild-type (WT) and the *osrrm* mutant grown under short day (SD) or long day (LD) conditions. Quantitative real-time PCR (qRT-PCR) analysis of flowering control genes. Leaf samples were collected every 4 h from 14-day-old plants grown under SD conditions and from 30-day-old plants grown under LD conditions. The rice *UBQ10* gene was used as an internal control for normalization of gene expression. White and black bars at the bottom indicate daylight and darkness, respectively. Three biological replicates were performed, with three technical replicates for each. Bars indicate SD. *p < 0.05, **p < 0.01 relative to WT.

Because *osrrm* plants exhibit a late-flowering phenotype, we also analyzed the expression of pivotal flowering control genes under long-day or short-day conditions (Figure 2). The expression of each gene was assayed every 4 h throughout two photoperiodic cycles. Under short-day conditions, compared with the WT, transcripts of *Heading date 3a* (*Hd3a*) and *RICE FLOWERING LOCUS T 1* (*RFT1*), which encode mobile flowering signals (Komiya et al., 2008), were drastically reduced in the *osrrm* mutant; the expression peaks for *Heading date 1* (*Hd1*), an ortholog of the *Arabidopsis* flowering time gene *CONSTANS* (*CO*) (Yano et al., 2000), occurred earlier; *O. sativa GIGANTEA* (*OsGI*), which controls the expression of *CO* (Hayama et al., 2003), was slightly upregulated at several time points; *Early heading date 1* (*Ehd1*), which controls the expression of *Hd3a* (Doi et al., 2004), was downregulated in the light and upregulated in the dark; there was no difference in the expression of *grain number*, *plant height*, *and heading date 7* (*Ghd7*), which inhibits the expression of *Ehd1* (Xue et al., 2008). In comparison, under long-day conditions, both *Hd3a* and *RFT1* were significantly downregulated; the expression pattern of *Ehd1* was essentially the opposite of that observed in the WT, and there were no significant differences in the expression of *Hd1*, *OsGI*, and *Ghd7* between the *osrrm* mutant and WT (Figure 2). Based on this result, we speculated that *OsRRM* functions as a regulator of the flowering regulation networks, and it acts upstream of *OsGI*, *Hd1*, and *Ehd1*, by which it adjusts the transcript levels of *Hd3a* and *RFT1*. In contrast, *OsRRM* has no effect on the expression of *Ghd7*.

### The *osrrm* Mutation Alters the Starch Content and the Fine Structure of Amylopectin

In cereals, sugars are primarily transported to sink reserve organs, such as seeds, and starch is the unique storage form for reserve sugars. Starch consists of two types of α-D-glucosyl homopolymers: the mostly linear amylose and the extremely branched amylopectin. The seeds of the *osrrm* mutant are smaller than in the WT, which may indicate altered storage products. To test this hypothesis, starch content and the chain length distribution of amylopectin were analyzed in the *osrrm* and WT seeds.

**FIGURE 3:**
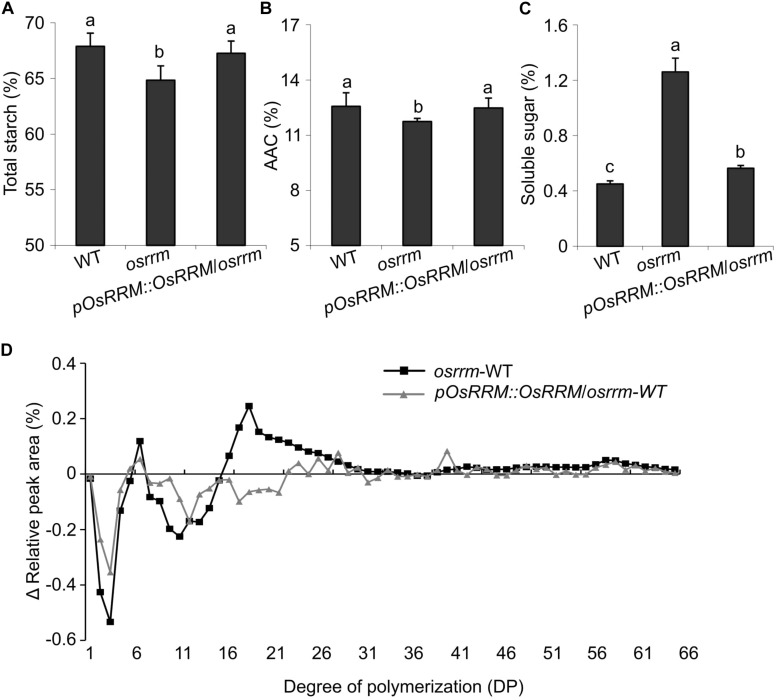
Changes in starch content and the fine structure of amylopectin in the *osrrm* mutant. **(A–C)** Total starch content **(A)**, apparent amylose content (AAC) **(B)**, and soluble sugar content **(C)** in endosperm. The different letters in panels **(A–C)** indicate statistically significant differences analyzed with one-way ANOVA followed by Tukey’s test (*p* < 0.05). **(D)** The differences in amylopectin chain lengths between *osrrm* and wild type (WT) and between *pOsRRM::OsRRM*/*osrrm* and WT. The distribution of chain lengths of total polysaccharide extracted from the endosperm was determined by high-performance anion-exchange chromatography with pulsed amperometric detection (HPAEC-PAD). The measurements were repeated three times with similar results. For all variables with the same letter or overlaps with the same letter (such as “a” and “ab”), the difference between the means is not statistically significant. In contrast, if two variables have different letters, they are significantly different.

Total starch content was significantly decreased in *osrrm* seeds (Figure 3A). In comparison, there was no statistical difference between WT and *osrrm* in terms of apparent amylose content (AAC) (Figure 3B), but the soluble sugar content showed a dramatic increase from 0.45% in WT to 1.25% in *osrrm* (Figure 3C). The mutant phenotypes could be reversed by expression of the WT OsRRM allele (Figures 3A–C).

As for amylopectin structure, the ratio of short chains was notably reduced, while the ratio of long chains was increased in *osrrm* seeds compared with WT. In particular, the proportion of short chains with degrees of polymerization (DP) of 6–9 and 12–18 was markedly decreased in *osrrm*, whereas the proportions of long chains with 40 < DP < 67 were increased. The proportion of intermediate-length chains (20 < DP < 32) was also dramatically increased in *osrrm* seeds (Figure 3D). The amylopectin chain length distributions were shifted toward that of the WT in *pOsRRM::OsRRM/osrrm* seeds (Figure 3D). These data imply that the synthesis rate of short-chain-length amylopectin is reduced in the *osrrm* mutant; this resembles the pattern seen in the *amylose-extender* (*ae*) mutant that carries a mutation in the gene encoding starch branching enzyme IIb (*SBEIIb*) (Nishi et al., 2001). Thus, in addition to shoot architecture and flowering time control, *OsRRM* may also have a function in preserving soluble sugar levels and in the synthesis of short branched amylopectin chains in the endosperm.

Starch synthesis-associated proteins such as granule-bound starch synthase 1 (GBSS1) and ADP-glucose pyrophosphorylase (AGPase) control amylose synthesis; AGPase, starch synthases (SSs), SBEs, and debranching enzymes (DBEs) such as isoamylase 1 (ISA1) control amylopectin synthesis; starch phosphorylase (SP) catalyzes both the biosynthetic and degradative reactions for α-1,4-linked glucan chains (Tetlow and Emes, 2017); and mutations in these genes alter the AAC and amylopectin structure (James et al., 2003). Thus, we examined the effect of the *osrrm* mutation on the expression of these genes in the endosperm of immature seeds 7 days after pollination (7 DAP). Western blot analysis showed that, except for slight decreases in SBEIIb and ISA1 in the *osrrm* mutant, the amounts of the AGPase, OsGBSS1, SSI, SSIIa, SBEI, and SP proteins in the *osrrm* mutant were comparable with those in WT (Supplementary Figure 4). This further indicates that the sugar reassignment in the *osrrm* mutant is not a consequence of altered gene expression or the levels of the enzymes that mediate starch synthesis.

### Sugar Transport Is Inefficient in the *osrrm* Mutant

**FIGURE 4:**
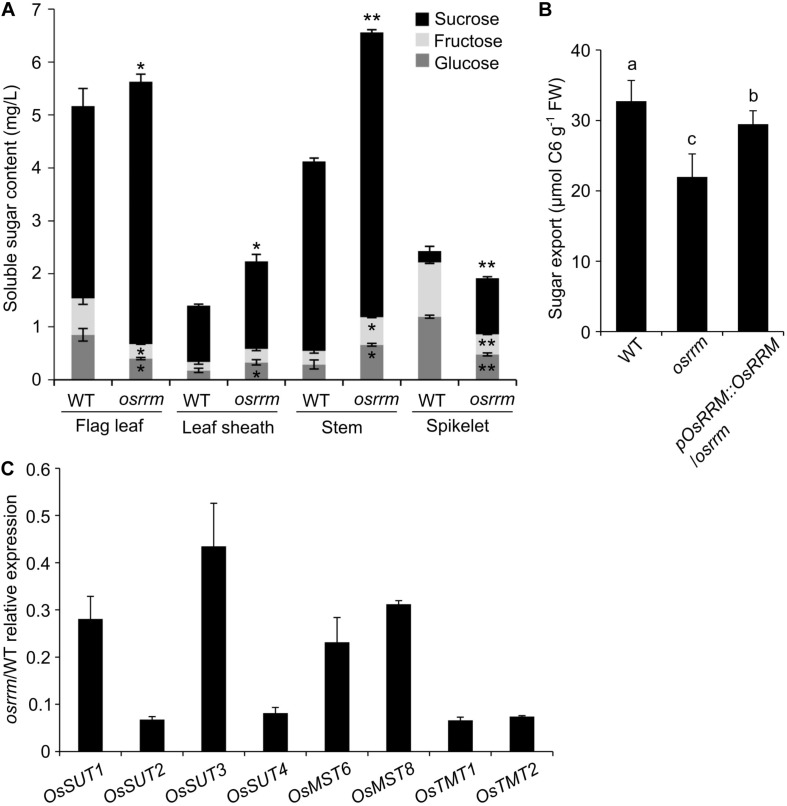
Decreased sugar export activity in the *osrrm* mutant. **(A)** The contents of three soluble sugars (sucrose, glucose, and fructose) in the flag leaf, flag leaf sheath, stem, and spikelet in the *osrrm* mutant and wild type (WT). *P* values (one-way ANOVA and Tukey’s post hoc), * < 0.05; ** < 0.01 relative to the values of WT. **(B)** Export of soluble sugars from the mature leaf blades of 4-week-old plants detached at the end of the day and incubated in 15 mM ethylenediamine tetraacetic acid (EDTA) solution for 16 h in the dark. Bars indicate standard deviations. FW, fresh weight. The different letters indicate statistically significant differences analyzed with one-way ANOVA followed by Tukey’s test (*p* < 0.05). **(C)** Expression of sugar transporter genes in stems of the *osrrm* mutant and WT. Stems were collected from 60-day-old WT and *osrrm* plants grown under short day (SD) conditions. Data shown are the relative expression levels of eight genes in the *osrrm* mutant plants against the expression in WT plants. Three biological replicates were performed with similar results. For all variables with the same letter or overlaps with the same letter (such as “a” and “ab”), the difference between the means is not statistically significant. In contrast, if two variables have different letters, they are significantly different.

We propose two hypotheses to explain the observed changes in starch in the *osrrm* mutant: (1) the shortage of monosaccharide (glucose, fructose) or disaccharide (sucrose) sugars, the primary donors for starch synthesis, or (2) decreased conversion efficiency from the donors to the final polysaccharide chains. However, the AAC was essentially unchanged (Figure 3B), and there was an almost three-fold increase in the soluble sugar content (from 0.45 to 1.25%), while the total starch content decreased by only 3% (from 67.8 to 64.8%) in the *osrrm* mutant (Figures 3A,C), meaning that the conversion of soluble sugars to polysaccharides was only mildly affected, if at all. To test hypothesis #1, we measured the contents of the soluble sugars, which include glucose, sucrose, and fructose, the principal sugars for long-distance transport *via* phloem sieve cells, in flag leaves, leaf sheaths of flag leaves, and stems. Our data showed that the sucrose, glucose, and fructose contents were all significantly increased in *osrrm* leaf sheaths and stems compared with WT (Figure 4A). In contrast, in the flag leaves and spikelets, along with the apparent increase in sucrose content, the contents of both glucose and fructose showed drastic decreases (Figure 4A).

The net accumulation of sugars in plants occurs during the day (in the light), while the net degradation of stored carbohydrate occurs in the dark during the night (Eom et al., 2011). We predicted that the enrichment of sucrose in the leaves might result from a reduced rate of transfer of sucrose to the sink tissues in the *osrrm* mutant. The sugar export was determined by measuring the soluble sugar levels in the exudates from detached mature leaf blades from the *osrrm* mutant and WT during the dark phase. As shown in Figure 4B, WT leaves exported 32.7 μmol C6 units/g FW soluble sugar, while *osrrm* leaves showed a decrease of ∼33% (22.0 μmol C6 units/g FW). The decreased sugar export in *osrrm* was complemented by introducing the WT *OsRRM* gene (Figure 4B).

Because the soluble sugar content differs significantly in *osrrm* plants compared to WT (Figures 3C, 4A), sugar export ability is hampered (Figure 4B) and the polysaccharide composition and structure are altered in the *osrrm* mutant (Figures 3A,D). No differences were found in the expression of starch synthesis-associated genes between the *osrrm* mutant and WT (Supplementary Figure 4), so it is highly likely that the difference in starch contents results from changes in sugar allocation caused by altered sugar transport. Because the stem is the vital hub for sugar transport from the leaves to sink tissues and sugar transporters mediate the transport of sugar from leaves to stem and from stem to seeds, we further examined the mRNA levels of sugar transport-related genes including *OsSUT 1-4*, the monosaccharide transporters *OsMST6* and *8*, and *OsTMT1* and *2* in stems of *osrrm* and WT plants at the beginning of the heading stage. We found that the relative abundance of *OsSUT1*-*4*, *OsMST6*, *OsMST8*, *OsTMT1*, and *OsTMT2* transcripts were all reduced, and the transcript levels of *OsSUT2*, *OsSUT4*, *OsTMT1*, and *OsTMT2* were affected the most (Figure 4C). Therefore, we hypothesize that the reduced transcription of these genes contributes to the phenotypes observed in the *osrrm* mutant, and when these sugar transporter genes were mutated, the plants phenocopied *osrrm* (Wormit et al., 2006; Wingenter et al., 2010; Eom et al., 2011).

### Expression Pattern of *OsRRM*

**FIGURE 5:**
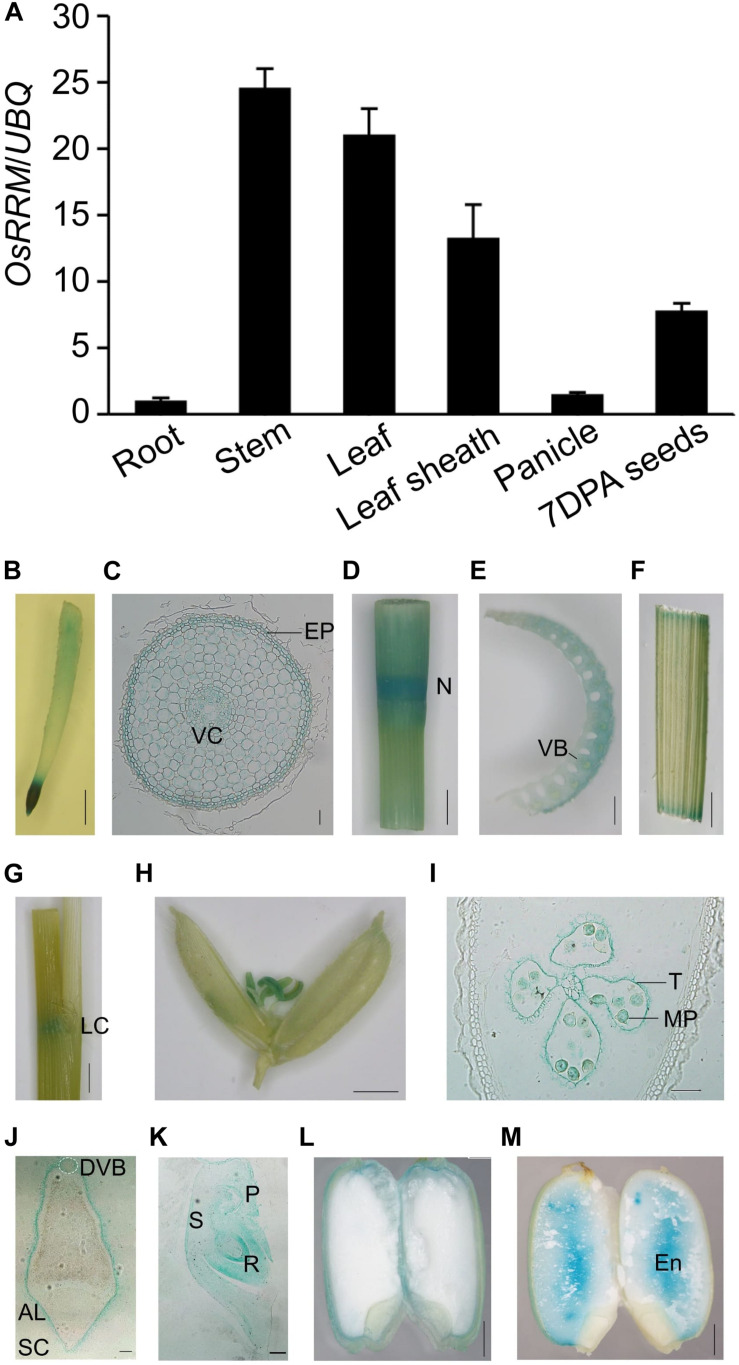
Expression pattern of *OsRRM* in rice. **(A)** Quantitative real-time PCR (qRT-PCR) analysis of *OsRRM* (*n* = 4 biological replicates). Total RNA was isolated from the roots (7-day-old seedlings), stems, leaf blades, leaf sheaths (before heading), panicles (before pollination), and 7 days after pollination (DAP) immature seeds. The expression of *UBQ10* was used as an internal control for normalization. Three biological replicates were performed with three technical replicates for each. Bars indicate standard deviations (SD). **(B–M)** Histochemical localization of GUS expression in six rice tissues. **(B,C)** Root; **(D,E)** stem; **(F,G)** leaf and leaf sheath; **(H)** spikelet before pollination; **(I)** stamen; **(J–M)** immature seed, 5 DAP **(J)**, 7 DAP **(K)**, 9 DAP **(L)**, 16 DAP **(M)**. Cross sections of root **(C)**, stamen **(I)**, and 5 DAP immature seed **(J,K)**, longitudinal section of embryo of 7 DAP seed. AL, aleurone layer; DVB, dorsal vascular bundle; En, endosperm; EP, epidermis; LC, leaf collar; MP, mature pollen; N, node; P, plumule; R, radicle; S, scutellum; SC, seed coat; T, tapetum; VC, vascular column. Scale bars, 5 mm for panels **(B,D,F,G,H)**, 50 μm for panels **(C,I,K)**, 1 mm for panel **(E)**, 200 μm for panel **(J)**, and 2 mm for panels **(L,M)**.

*OsRRM* was originally described as a gene that is expressed specifically in the endosperm (Chen et al., 2007), which makes it difficult to explain how *OsRRM* controls the expression of genes involved in flowering time and sugar transport, two events that occur well before endosperm development. To reinvestigate the spatiotemporal expression pattern of *OsRRM*, qRT-PCR assays were performed. The *OsRRM* transcription levels were analyzed in the following tissues: roots of 7-day-old seedlings, stems, leaves, and leaf sheaths harvested before heading, panicles harvested before pollination, and 7 DAP immature seeds (Figure 5A). We found that the highest level of *OsRRM* expression occurs in stems and leaves, and that *OsRRM* expression is higher in leaf sheaths and immature seeds than it is in panicles and roots.

We also generated transgenic plants expressing *pOsRRM*::*OsRRM* gDNA-GUS and performed histochemical GUS analysis. Ten independent transgenic plantlets were generated, and no variation was observed in the GUS expression pattern among them besides variations in expression level. GUS activity was detectable in roots (Figures 5B,C), stems (Figures 5D,E), leaves and leaf sheaths (Figures 5F,G), stamens (Figures 5H,I), and immature seeds (Figures 5J–M). In roots, GUS activity was detected in the epidermis and the vascular column of the root elongation zone (Figure 5C) and also in the tapetum and mature pollen in stamens (Figure 5I). Most interestingly, GUS activity was observed in the aleurone layer and embryo, but not in the dorsal vascular bundle at the early starch-filling stage (5–9 DAP) in seeds (Figures 5J–L), and staining was only detectable in the endosperm of mature seeds (16 DAP) (Figure 5M). Notably, although *OsRRM* expression could be detected in almost all tissues, higher expression levels were observed in internodes, which partly resembles the expression profile of *OsDOF11* (Wu et al., 2018) in pollen, which partly resembles the expression of *OsSUT1* (Hirose et al., 2010) in immature seeds, which is similar to the expression of the sugar transporter gene *OsSUT2* (Eom et al., 2011) and *OsSWEET11* (Ma et al., 2017).

### OsRRM Binds Directly to the Sugar Transporter Gene mRNAs

**FIGURE 6:**
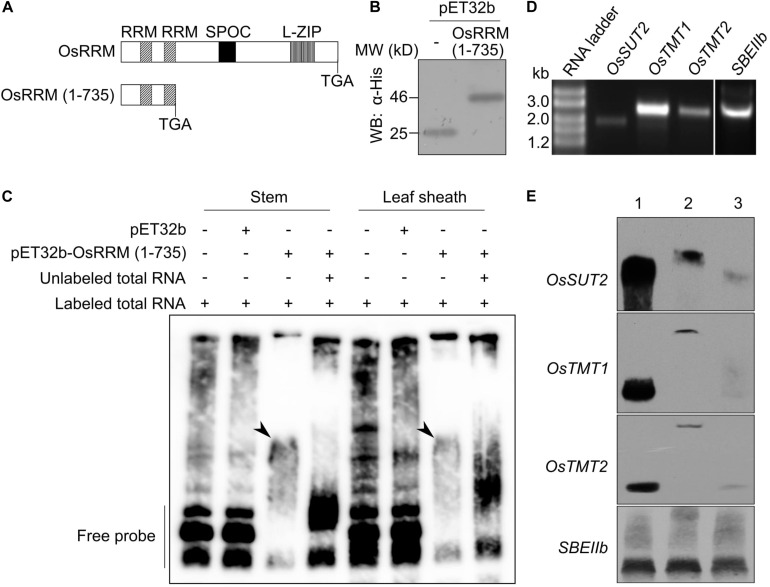
The truncated OsRRM protein binds specifically to messenger RNAs (mRNAs) of *OsSUT2*, *OsTMT1*, and *OsTMT2*. **(A)** Schematic diagram showing the locations of functional domains in the OsRRM protein and the truncated OsRRM (1-735) protein used in this study. **(B)** Protein gel Western blot (WB) analysis of the purified pET32b and pET32b-OsRRM (1-735) using an anti-His monoclonal antibody (Abmart). For pET32b-OsRRM (1-735), the coding region of the truncated OsRRM protein was fused in-framed with the 6× His-tag. **(C)** RNA electrophoretic mobility shift assay (REMSA) of pET32b-OsRRM (1-735) with rice total RNA. Total RNA was isolated from stems and leaf sheaths of ZH11 before heading and was labeled with biotin. Unlabeled stem and leaf sheath RNA (100-fold) were used for the competition assay. pET32b was the negative control. Arrowheads indicate the positions of RNA–protein complexes. **(D)** Gel electrophoresis of *in vitro* transcribed full-length mRNAs of *OsSUT2*, *OsTMT1*, *OsTMT2*, and *SEBIIb*. RNA ladder size markers (Thermo Fisher Scientific). **(E)** REMSAs using full-length mRNAs of *OsSUT2*, *OsTMT1*, *OsTMT2*, and *SBEIIb*, and pET32b-OsRRM (1-735). Lane 1, biotin-labeled RNA of interest; Lane 2, incubation of biotin-labeled RNA with pET32b-OsRRM (1-735) protein; Lane 3, incubation of biotin-labeled RNA with pET32b-OsRRM (1-735) protein and unlabeled RNA of interest as competitor. The experiments were performed twice for panels **(C,E)** with similar results.

OsRRM was shown to be predominantly localized to the nucleus (Chen et al., 2007), where it may confer its function by specifically binding to its target RNAs. To test this hypothesis, and because the full-length OsRRM could not be expressed in *E. coli*, we used a truncated form of OsRRM [OsRRM (1-735)] together with biotin-labeled total RNA isolated from stems or leaf sheaths to perform REMSAs. OsRRM (1-735) consists of the coding region of OsRRM from the start codon to the second RRM domain (Figure 6A), and we speculated that it would be capable of RNA binding. The control protein, pET32b, which contains only the 6× His tag, together with pET32b-OsRRM (1-735), was expressed in *E. coli* and further purified (Figure 6B). The biotin-labeled RNA was incubated with or without OsRRM (1-735), followed by electrophoresis under native conditions. RNA band shift was only observed when the labeled RNA was incubated with OsRRM (1-735), but not with control protein, pET32b (Figure 6C). The mobility shift could be abolished by further addition of unlabeled RNA (Figure 6C). Thus, this experiment demonstrated that OsRRM is a functional RNA-binding protein.

Because *osrrm* mutant plants phenocopy several monosaccharide or disaccharide transporter mutants, and the transcript levels of these sugar transport-related genes were reduced in the *osrrm* mutant (Figure 4C), we considered that *OsRRM* may be involved in posttranscriptional control of these sugar transporter genes. Also, the expression patterns of *OsDOF11* (Wu et al., 2018) and *NF-YB1* (Bai et al., 2016) do not fully overlap with that of *OsRRM*, especially in the roots, leaf collar, and seeds. In addition, the *osrrm* mutation causes decreased *OsSUT2* transcription, which is not phenocopied by mutation in either of those two transcription factor genes; therefore, we think that OsRRM, by directly binding to the pre-mRNA or mature mRNAs transcribed from those sugar transporter genes, controls their transcript levels. In order to investigate this, we carried out REMSAs with OsRRM (1-735) and the full-length *OsSUT2*, *OsTMT1*, and *OsTMT2* mRNAs, with and *SBEIIb* mRNA as the control (Figures 6D,E). As shown in Figure 6E, OsRRM (1-735) bound to biotin-labeled *OsSUT2*, *OsTMT1*, and *OsTMT2*, and unlabeled mRNA competed for the binding. In contrast, OsRRM (1-735) did not bind *SBEIIb* mRNA (Figure 6E). Thus, OsRRM may positively regulate the mRNA levels of these three sugar transporter genes at the posttranscriptional level.

## Discussion

In this study, we determined the RNA-binding capability of OsRRM to sugar transporter gene mRNAs, by which OsRRM calibrates their transcript levels to fine-tune sugar transport on the source-to-sink tissue route. The changes in sugar allocation and the coincident changes in sugar signaling contribute to the dwarf stature, late flowering, smaller seeds, and the altered soluble sugar content and amylopectin structure of the *osrrm* mutant. Thus, we may have paved the way for identifying genes that generally control sugar transport by monitoring posttranscriptional control.

Even though we have demonstrated the RNA-binding capability of OsRRM to sugar transporter gene mRNAs, how these transcripts are affected by the binding remains to be determined. OsRRM could be involved in preventing nonsense-mediated mRNA decay, maintaining RNA stability, and facilitating RNA transport to the nuclear pore or it may function in posttranscriptional processing. These are all open questions for us to further unravel the biological functions of OsRRM.

*OsRRM* expression was observed in all tissues that we analyzed, but with higher expression in stems, especially in the internode, and in pollen and immature seeds, although it was first described as an endosperm-specific gene (Chen et al., 2007). This discrepancy is possibly due to the loss of the *OsRRM* coding region in the promoter trap system used by Chen et al. (2007). It has been shown that gene coding regions may be required for promoter activity or microRNA (miRNA) targeting in some genes (Juarez et al., 2004; Jang et al., 2014). *OsRRM* has an expression profile that is to some extent similar to that of *OsSUT1* and *OsSUT2*. Because co-expressed genes can have related functions (Niehrs and Pollet, 1999), as exemplified by the fact that starch synthesis-associated genes are all specifically expressed in the endosperm, we predicted that OsRRM is associated with sugar transport.

The phenotype of *osrrm* mutant plants resembles the late-flowering phenotype of *OsRRMh* knockdown plants (Liu and Cai, 2013), and *OsRRM* may act upstream of *Ehd1*, but it is not linked to *Ghd7* in the flowering time control network. We know that efficient sugar retention is a necessity for optimal plant growth and productivity, and sugar transport is obstructed in the *osrrm* mutant. As signaling molecules, sucrose and trehalose 6-phosphate influence *Arabidopsis* flowering by modulating the expression of *LEAFY* (Ohto et al., 2001) or by affecting the miRNA156-mediated regulation of *FT* expression (Lastdrager et al., 2014). Thus, it would be reasonable to deduce that OsRRM plays roles in the rice flowering time control network by adjusting the transport of soluble sugars, which further modulates sugar signaling transduction.

In the *osrrm* mutant, along with the impaired sugar export, the contents of soluble sugars (sucrose, glucose, and fructose) varied in the tissues that lie along the sugar translocation route. This is a result of a reduction in modulation by OsRRM of the posttranscriptional level of the sugar transporter genes because the transcripts of most of them were significantly reduced in the *osrrm* mutant, making OsRRM a positive posttranscriptional regulator and directly linking OsRRM with sugar transport. On the contrary, however, the Spen protein in animal cells, a homolog of OsRRM, is a transcriptional repressor (Chu et al., 2015). This is worth investigating because similar to Spen, OsRRM also contains a SPOC domain, which facilitates the associations between the Spen protein and a transcriptional co-repressor in the transcriptional repression complex (Ariyoshi and Schwabe, 2003). The interactome of the OsRRM SPOC domain may shed some light on this discrepancy.

For most of the sugar transporter genes and other genes recruited in sugar metabolism or sensing, either their expression or activity is controlled by sugar levels (Lu et al., 2007; Wingenter et al., 2010). In addition, ABA may regulate apoplastic sugar transport by influencing the expression of sugar transporter genes (Oliver et al., 2007). Because the expression of *OsRRM* is directly associated with sugar content and sugar transport, we deduced that there may be a feedback effect of altered sugar and (or) ABA content on *OsRRM* expression. We therefore tested the effects of soluble sugars on *OsRRM* expression. ZH11 seedlings were treated with only 200 mM sucrose, glucose, or mannitol, or 200 mM mannitol together with 100 μM ABA, or 200 mM sucrose together with 100 μM ABA. Subsequent RT-PCR analyses showed that, unlike the expression of the known sugar-inducible genes such as *OsTMT1* and *OsTMT2* (Cho et al., 2010) and glucose-repressed genes such as *OsSUT2* (Eom et al., 2011), *OsRRM* expression was unchanged when compared with the mannitol only control treatment (Supplementary Figure 5). In addition, the expression of *OsRRM* was unaffected by exogenous ABA treatment (Supplementary Figure 5). Although we have demonstrated that *OsRRM* expression is not affected by sugar content and ABA level, we cannot exclude the possibility that sugars and/or ABA could affect OsRRM stability and/or its ability to bind RNA. The retarded development observed in *osrrm* mutant plants could possibly be reversed under these conditions and may resemble the complementation of the *Arabidopsis sbp* mutant by exogenous sucrose (Liu et al., 2012).

RNA-binding proteins can maintain mRNA stability by binding to the target sequence [CDS or untranslated regions (UTRs)] (Brummer and Hausser, 2014; Goodarzi et al., 2014). Regulatory RBPs, in most instances, cooperate or compete with miRNAs to regulate the destinies of multiple mRNAs with similar functions (Keene, 2007; Halbeisen et al., 2008), a principle that has been fully confirmed in the concept of the posttranscriptional RNA operon/regulon (Keene, 2014). This may explain why OsRRM binds to the transcripts of sugar transporter genes as a group. OsRRM shows specificity toward its target RNAs because it did not bind to *SBEIIb* mRNA. However, the way that OsRRM binds specifically to those transcripts is unknown. With that being said, we could not exclude the possibility that OsRRM, by directly binding to *OsDOF11* and (or) *NF-YB1*, controls the mRNA level of sugar transporter genes given that they share expression patterns in stems with *OsDOF11* (Wu et al., 2018) and in the aleurone layer with *NF-YB1* (Bai et al., 2016). RNA binding specificity is determined by consensus binding motif(s), which, if deduced for OsRRM, could trigger the identification of other target mRNAs (genes) of OsRRM. Identifying the consensus motif(s) for OsRRM-bound mRNAs is a considerable challenge. There are two different methods that could be applied to fulfill this goal: (1) using photoactivatable ribonucleoside-enhanced crosslinking and immunoprecipitation (PAR-CLIP) (Ascano et al., 2012) to first obtain the enriched sequence information in the WT compared with the *osrrm* mutant, followed by sequence alignment, and (2) carrying out yeast-three hybridization (Hook et al., 2005).

The *osrrm* mutation systematically affects growth and development in rice, which could explain why ectopic overexpression of *OsRRM* also causes retarded growth and dwarfing (Chen et al., 2007); as the sugar allocation balance between source and sink tissues is disrupted, more soluble sugars are pumped to sink tissues, thus retarding plant growth. Overexpressing *OsRRM* at the dorsal vascular bundle of seeds may increase the efficiency of seed filling and thus increase seed size, making this gene a potential target for metabolic engineering to increase food security.

## Data Availability Statement

The original contributions presented in the study are included in the article/Supplementary Material, further inquiries can be directed to the corresponding author/s.

## Author Contributions

JG and DL designed the study and wrote the manuscript. DL, LX, WW, SJia, and SJin performed the experiments. All authors discussed and interpreted the results.

## Conflict of Interest

The authors declare that the research was conducted in the absence of any commercial or financial relationships that could be construed as a potential conflict of interest.

## Footnotes

1 https://www.dna.affrc.go.jp

## Supplementary Material

Supplementary Figure 1 Isolation of the *osrrm* mutant allele.

Supplementary Figure 2 Cells are smaller in glumes of the *osrrm* mutant.

Supplementary Figure 3 Molecular characterization of the *osrrm* complemented line.

Supplementary Figure 4 Western blot (WB) analysis of eight starch synthesis-associated proteins in 7 DAP endosperms of WT and the *osrrm* mutant.

Supplementary Figure 5 *OsRRM* expression in ZH11 plants treated with exogenous sugars and (or) ABA.

Supplementary Table 1 Oligonucleotide primers used in this study.
