# Supplementary figures and images for: OsRRM, an RNA-Binding Protein, Modulates Sugar Transport in Rice (Oryza sativa L.)
Source: Front Plant Sci. 2020 Dec 8;11:605276. doi: 10.3389/fpls.2020.605276 (PMC7752781; doi:10.3389/fpls.2020.605276)

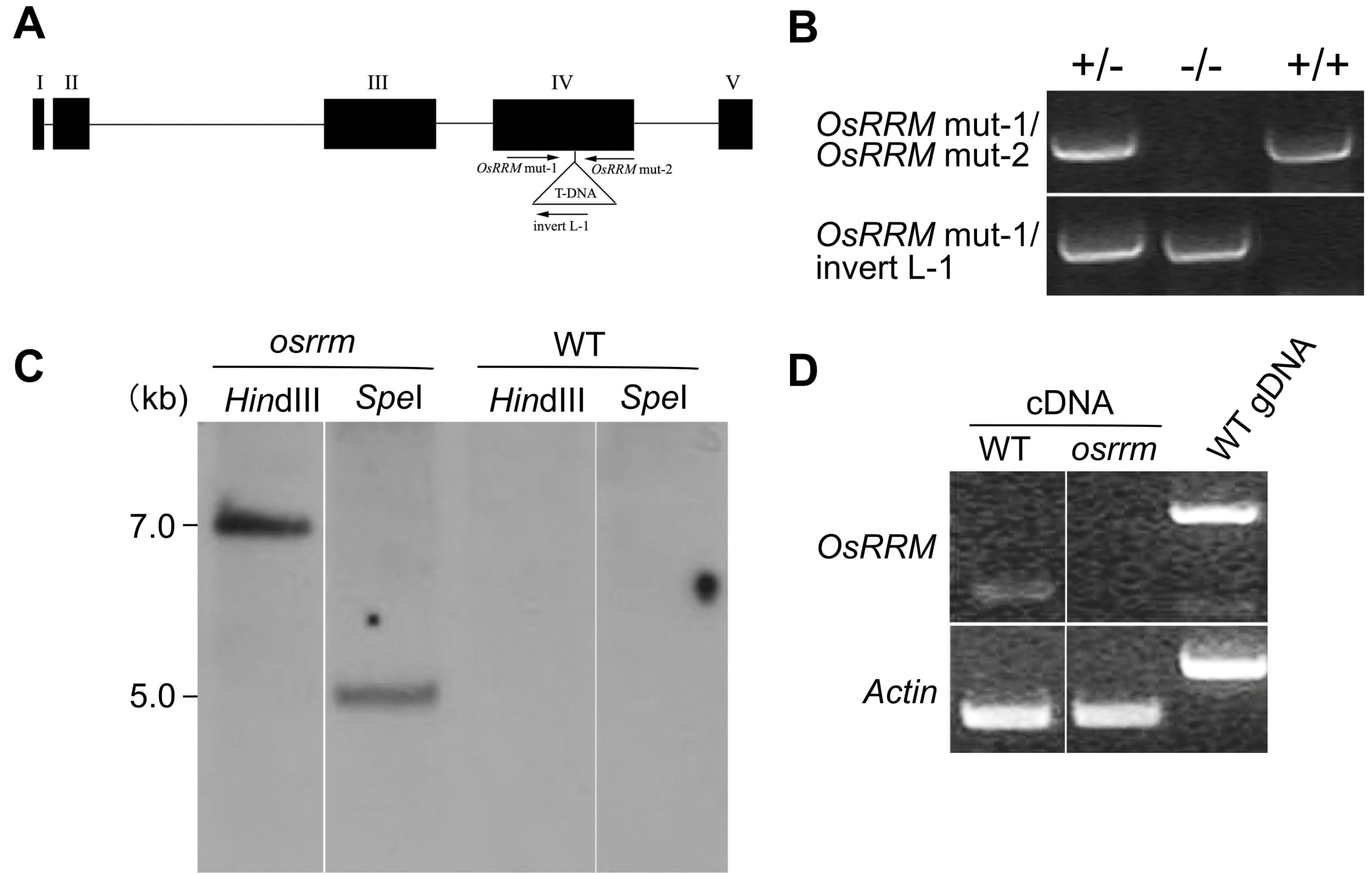

Supplement: Supplementary Figure 1 — Isolation of the osrrm mutant allele. [file Image_1.JPEG]

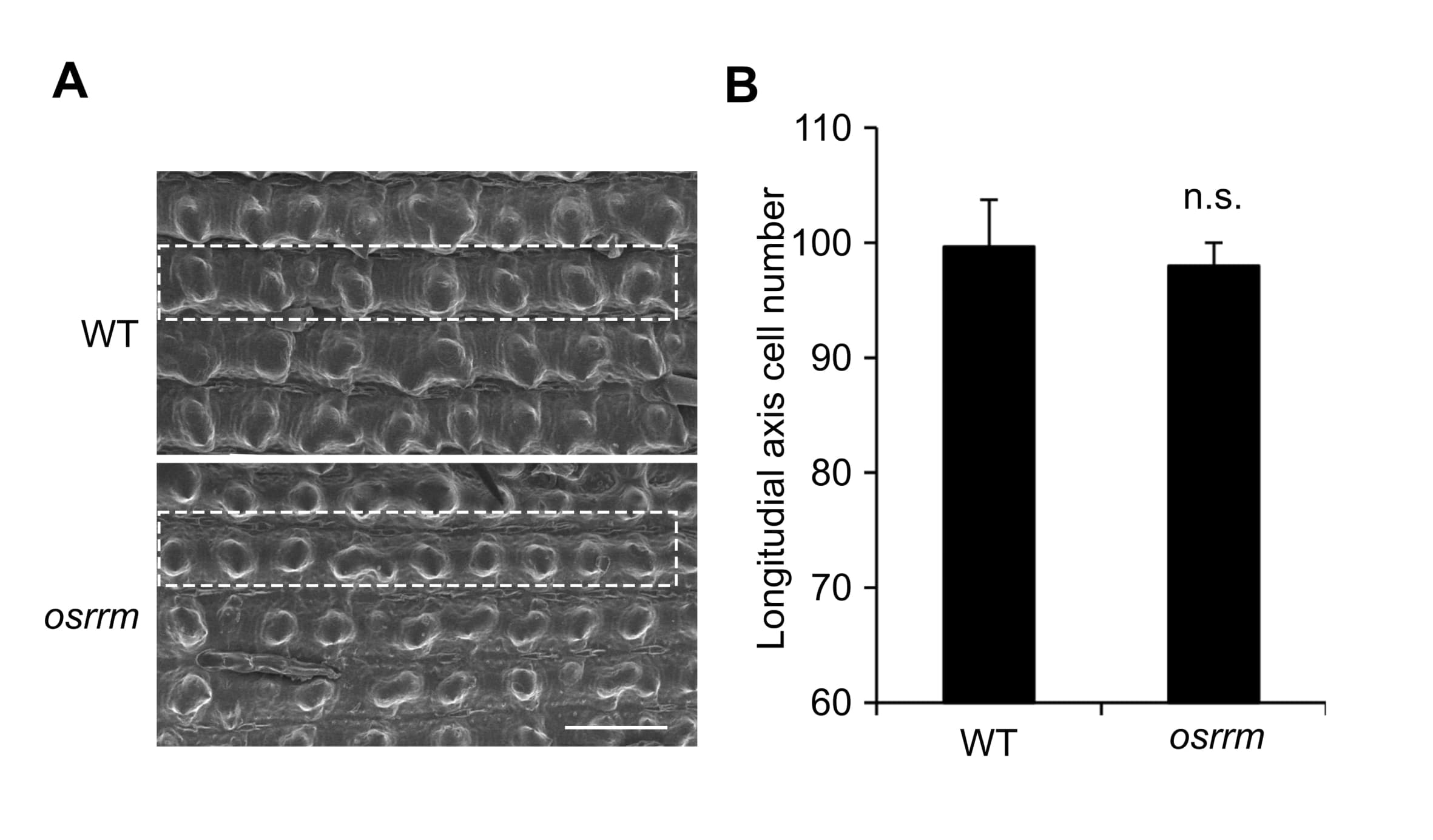

Supplement: Supplementary Figure 2 — Cells are smaller in glumes of the osrrm mutant. [file Image_2.JPEG]

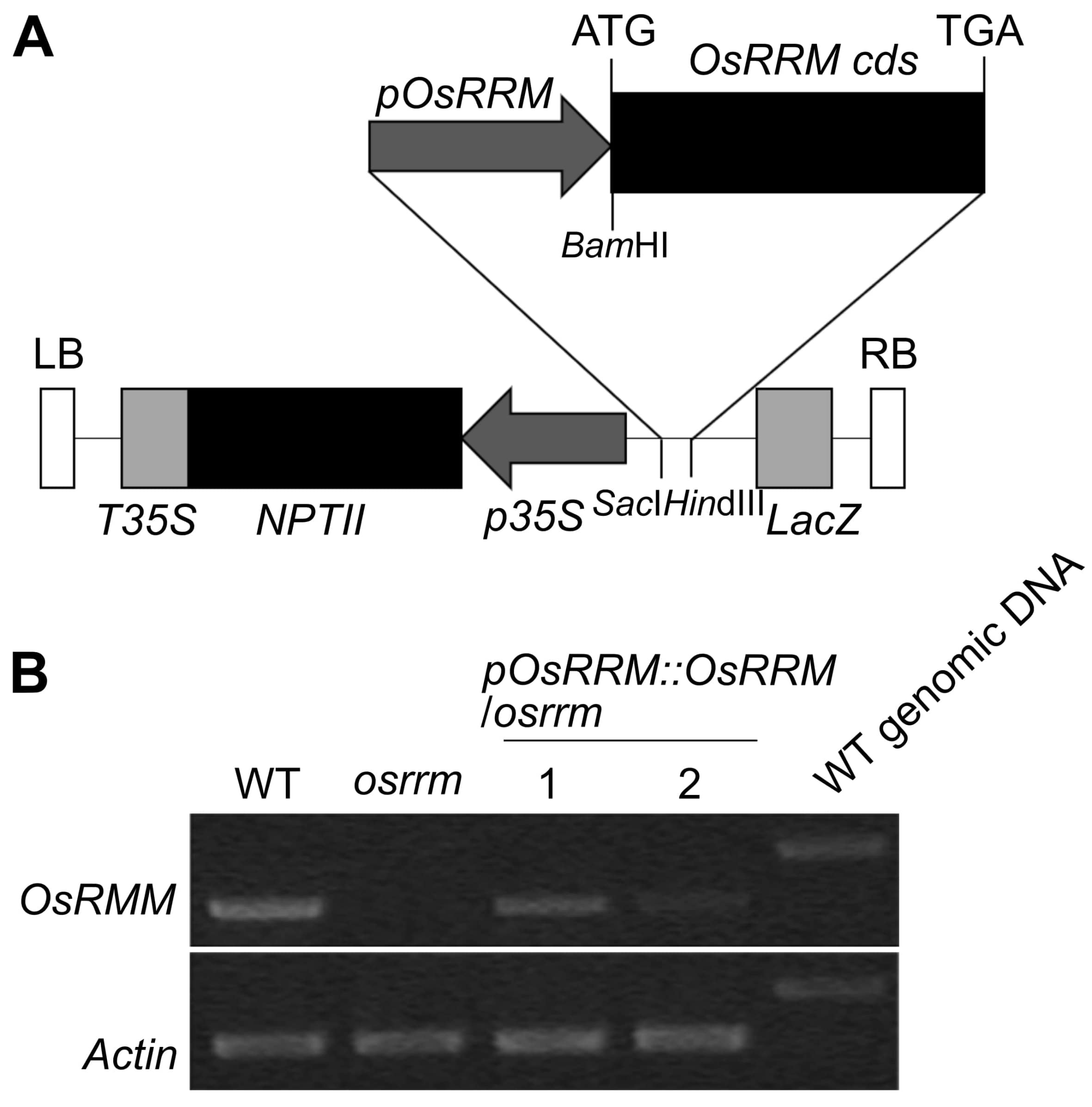

Supplement: Supplementary Figure 3 — Molecular characterization of the osrrm complemented line. [file Image_3.JPEG]

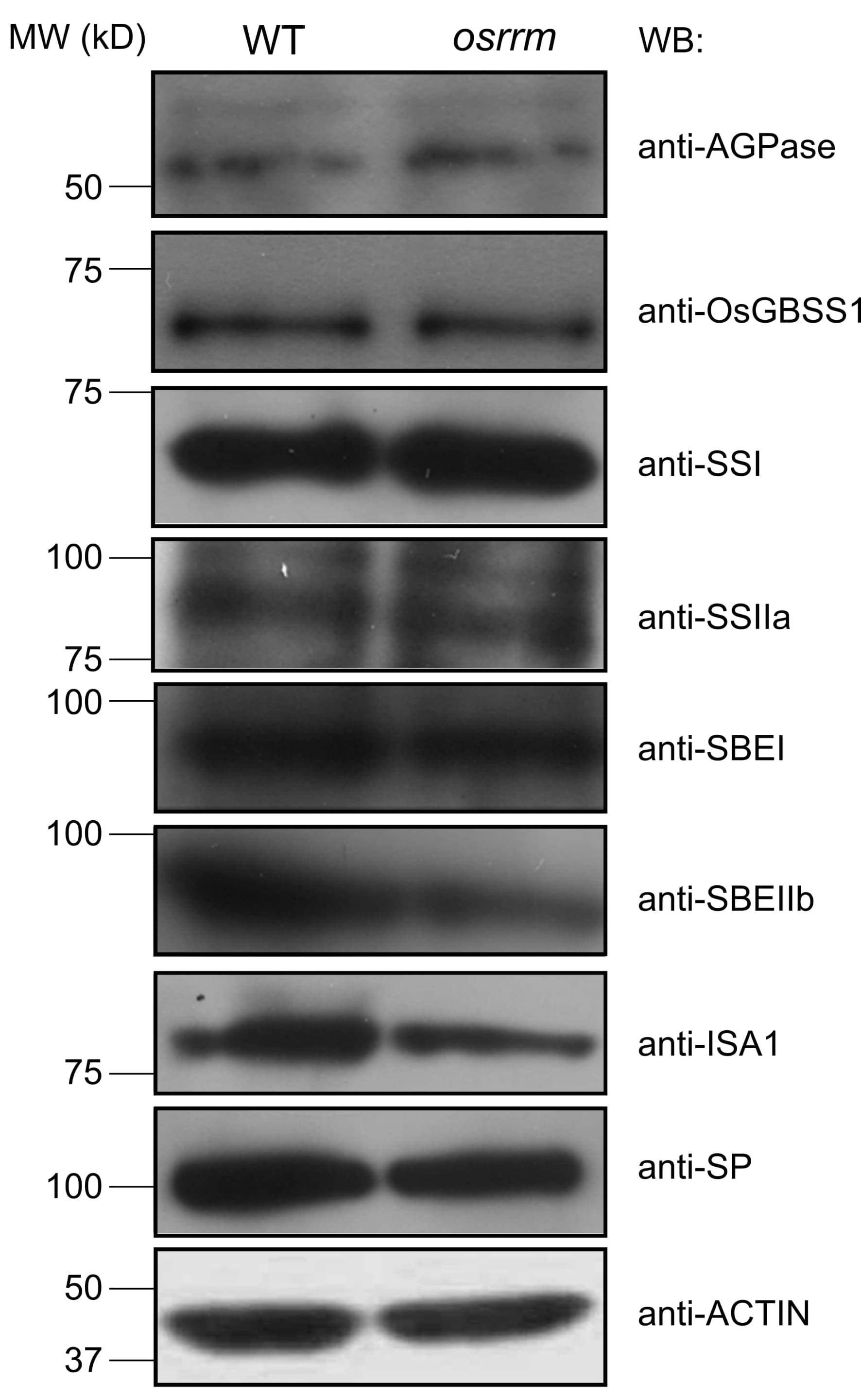

Supplement: Supplementary Figure 4 — Western blot (WB) analysis of eight starch synthesis-associated proteins in 7 DAP endosperms of WT and the osrrm mutant. [file Image_4.JPEG]

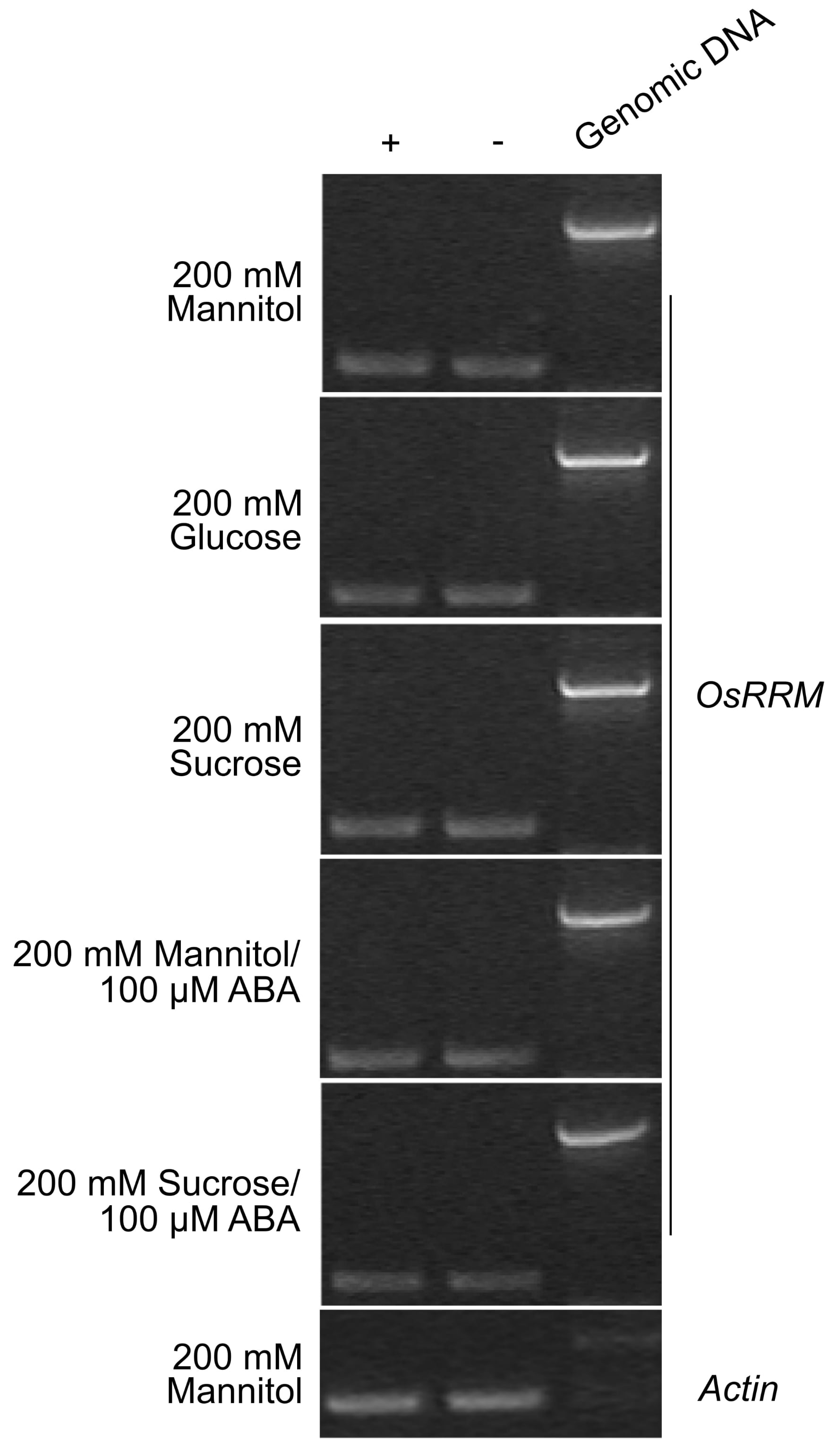

Supplement: Supplementary Figure 5 — OsRRM expression in ZH11 plants treated with exogenous sugars and (or) ABA. [file Image_5.JPEG]
