# Supplementary material for: OsRRM, an RNA-Binding Protein, Modulates Sugar Transport in Rice (Oryza sativa L.)
Source: Front Plant Sci. 2020 Dec 8;11:605276. doi: 10.3389/fpls.2020.605276 (PMC7752781; doi:10.3389/fpls.2020.605276)
Supplement: Supplementary Table 1 — Oligonucleotide primers used in this study. [file Data_Sheet_1.PDF]

**Supplementary Figures and Tables**

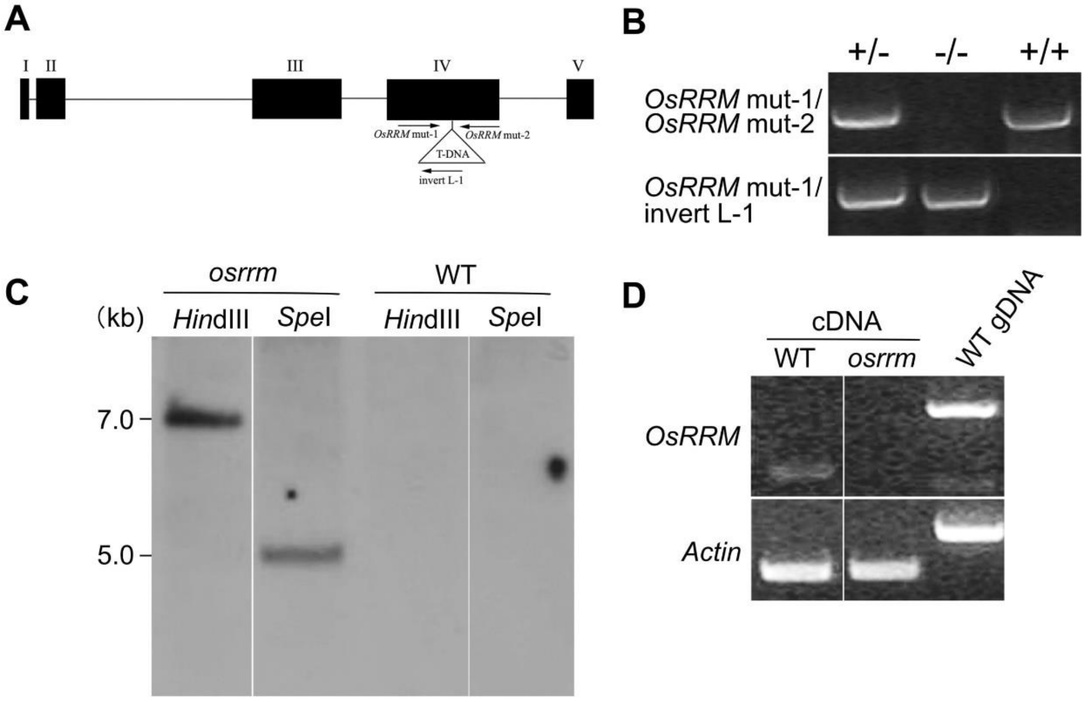


**Figure S1.** Isolation of the *osrrm* mutant allele. **(A)** Diagram showing the structure of the *OsRRM* gene and the T-DNA insertion site in the *osrrm* mutant allele. Numbered black squares indicate the exons, and the introns are indicated by lines. The stick-linked triangle shows the T-DNA insertion site in *osrrm*. Arrows show the position and polarity of the primers used in the genotyping. **(B)** Determination of zygosity at the *OsRRM* locus by genotyping. Primers used are indicated in **(A)**. +/- denotes a heterozygous (*OsRRM*/*osrrm*) mutant plantlet; -/-, homozygous (*osrrm*/*osrrm*) mutant plantlet; +/+, wild-type (*OsRRM*/*OsRRM*) plantlet. **(C)** Detection of T-DNA copy number in the *osrrm* mutant allele by Southern blotting. Genomic DNA isolated from homozygous *osrrm* or WT plants was digested with *Hin*dIII or *Spe*I, followed by agarose gel electrophoresis, transfer of the DNA to a nylon membrane, and hybridization with a biotin-labeled DNA fragment encompassing the *HPTII* coding sequence. **(D)** Expression analysis of *OsRRM* in nodes of WT and homozygous *osrrm* mutant plants by semi-quantitative RT-PCR. The expression of *Actin* was used as an internal control. The experiments were performed twice with similar results.

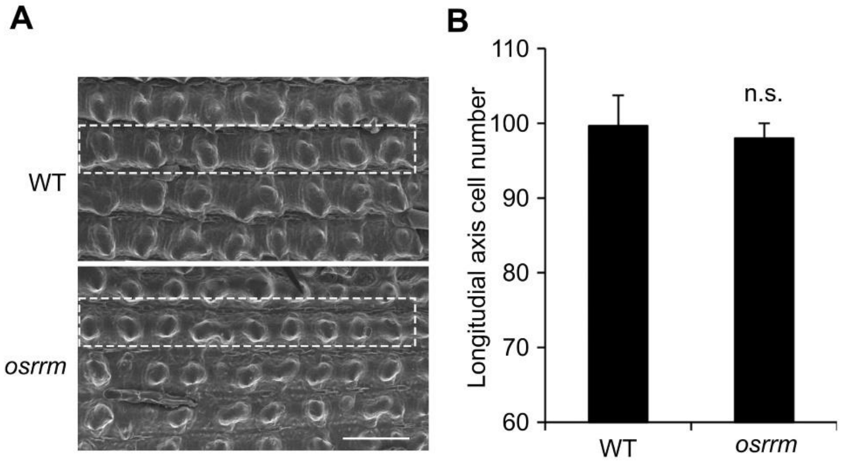


**Figure S2.** Cells are smaller in glumes of the *osrrm* mutant. **(A)** SEM of lemma cells in WT and the *osrrm* mutant. Note the number of cells in the regions outlined by a white dashed line. Scale bar, 100 µm. **(B)** The total number of lemma cells along the longitudinal axis of the glume was counted in the WT and the *osrrm* mutant. Experiments were performed twice with similar results. The data shown is the average of five glumes.

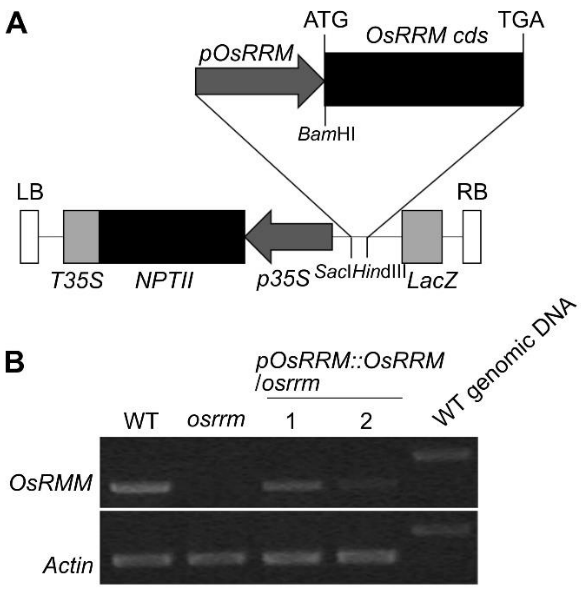


**Figure S3.** Molecular characterization of the *osrrm* complemented line. **(A)** Diagram of the T-DNA region

of the plasmid vector constructed to complement the *osrrm* mutant by expressing the WT *OsRRM* allele. Gray arrows and black, light gray, and white rectangles denote promoters, gene coding regions, terminators, and T-DNA borders, respectively. **(B)** Expression analysis of *OsRRM* in nodes from WT, homozygous *osrrm*, and *pOsRRM::OsRRM/osrrm* plants by semi-quantitative RT-PCR. The expression of *Actin* was used as an internal control.

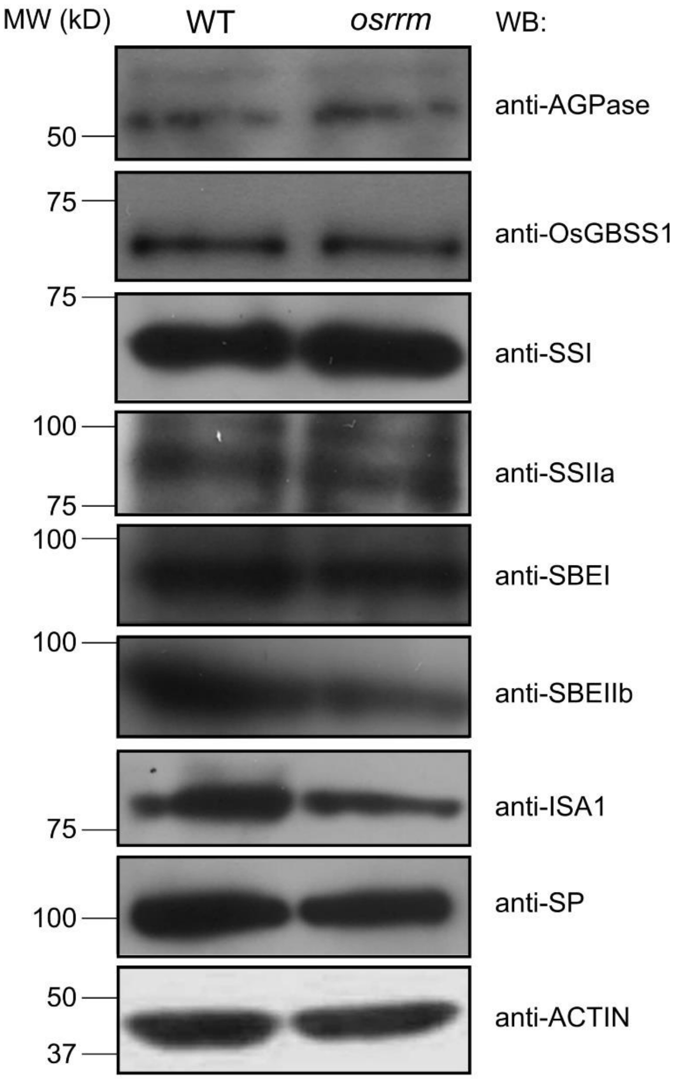


**Figure S4.** Western blot (WB) analysis of eight starch synthesis-associated proteins in 7 DAP endosperms of WT and the *osrrm* mutant. Total protein samples (20 µg) isolated from immature

endosperms of WT and *osrrm* grains were fractionated on 8% or 10% denaturing polyacrylamide gels, transferred to a PVDF membrane, and detected with anti-AGPase, -OsGBSS1, -SSI, -SSIIa, -SBEI, -SBEIIb, -ISA1, and -SP antibodies, respectively. The protein inputs were equilibrated by WB with the anti-ACTIN antibody (bottom). The experiments were performed twice with similar results.

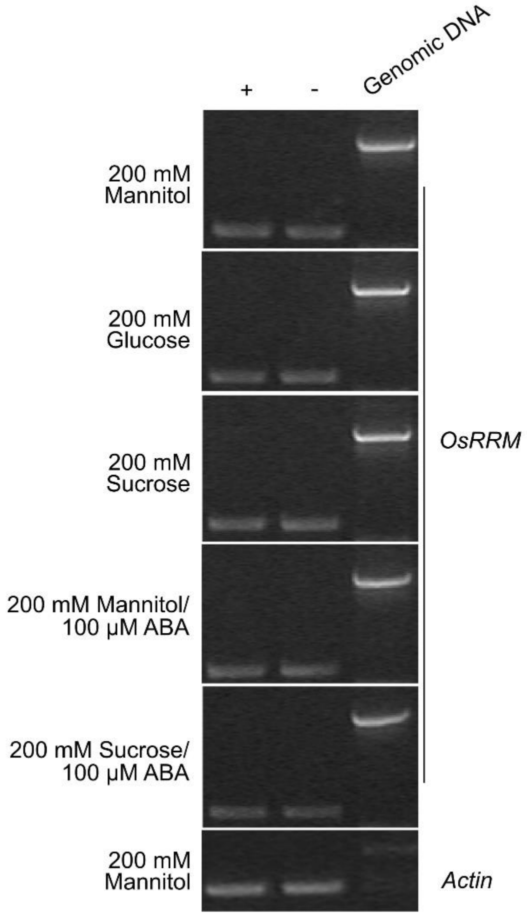


**Figure S5.** *OsRRM* expression in ZH11 plants treated with exogenous sugars and (or) ABA. "+" and "-" denote plants with or without exogenous treatments. Total RNA isolated from 5-day-old seedlings treated with sugar or ABA in $H_2O$ overnight was subjected to semi-quantitative RT-PCR analysis of *OsRRM* expression. The expression of *Actin* was used as an internal control. The experiments were performed twice with similar results.

**Table S1.** Oligonucleotide primers used in this study

| Primer Name | Primer Sequence (5'-3') | Description | Reference |
|---|---|---|---|
| *OsRRMp*-5' | GACCACCCGGGGATCCCTTCTGGCCACCAGG | Cloning *pORRM-OsRRM* | |
| *OsRRM*-3' | CCAAGCTTGCATGCCTGCAGTTTTTCCATCCTTGAG | | |
| *OsRRM* mut-1 | CATGAGGATCTAGATGCATTGAG | *osrrm* genotyping | |
| *OsRRM* mut-2 | GATTTCCCGAAGGATGCATAGG | | |
| inver L-1 | AGGCTTTGATAGTCACAGATCGA | | |
| *Hyg*-F | GCTTTCAGCTTCGATGTAGGAGG | *HPTII* probe | |
| *Hyg*-R | TTTCCACTATCGGCGAGTACTTC | | |
| *OsGI*_F | TGGAGAAAGGTTGTGGATGC | qRT-PCR | (Liu et al. 2013) |
| *OsGI*_R | GATAGACGGCACTTCAGCAGAT | | |
| *Ghd7*_F | AAATCCGGTACGCGTCCAG | | |
| *Ghd7*_R | GACATAGGTGGATGGCGGTG | | |
| *Hd1*_F | TCAG CAACAGCATATCT TTCTCATCA | | |
| *Hd1*_R | TCTGGAATTTGGCTATACTATCACC | | |
| *Ehd1*_F | GCGCTTCTGATTTCCTGC | | |
| *Ehd1*_R | CGGAATATGTGCTGCCAG | | |
| *Hd3a*_F | GCTAACGATGATCCCGAT | | |
| *Hd3a*_R | CCTGCAATGTATAGCATGC | | |
| *RFT1*_F | CGTCCATGGTGACCCAACA | | |
| *RFT1*_R | CCGGGTCTACCATCACGAGT | | |
| *RCN1*_F | GACCTGCGATCTTTCTTCAC | | |
| *RCN1*_R | CCGTTGTCCCAGGTATATCA | | |
| *OsSUT1*_F | TCATCCCTCAGGTGGTCATCG | qRT-PCR | (Hirose et al. 2010) |
| *OsSUT1*_R | CTTGGAGATCTTGGGCAGCAG | | |
| *OsSUT3*_F | TCCTCTTCGACACCGACTG | | |
| *OsSUT3*_R | CAGCACGATCGAGTTAAGGAG | | |
| *OsSUT4*_F | CGTTGTTCCGCAGATAGTAGTG | | |
| *OsSUT4*_R | GTGTTCTGCTCAGCCAAATCC | | |
| *OsSUT2*_F | TGTGGCAAAGAATATGGATTAT | qRT-PCR | (Eom et al. 2011) |
| *OsSUT2*_R | CTACCCAGTGACACAATAACCT | | |
| *OsMST6*_F | AATCTCGAGATGGCCGGCGGCGTGGTG | qRT-PCR | (Wang et al. 2008) |
| *OsMST6*_R | AATGGTACCGTTGGCGAGCTTGGCCGG | | |
| *OsMST8*_F | GTCCTCTTCAAGACCATCGGAT | qRT-PCR | (Zhang et al. 2010) |
| *OsMST8*_R | TACCAGTGCTTACCCCAGATC | | |
| *OsTMT1*_F | AGGTGATGTTCCTCCTGGTG | qRT-PCR | (Cho et al. 2010) |
| *OsTMT1*_R | TGCCCATTTTGGACCTTTAG | | |
| *OsTMT2*_F | GGTTGATCAATGGGATGAGG | | |

| | | | |
|---|---|---|---|
| *OsTMT2*_R | ATGAGCATGGTGTGCAATGT | | |
| *UBQ10*_F | TGGTCAGTAATCAGCCAGTTTGG | | |
| *UBQ10*_R | GCACCACAAATACTTGACGAACAG | | |
| *qOsRRM*_F | GATCGAGTTTTCCAAGGGGGA | | |
| *qOsRRM*_R | AAGGCTTCCCAGAGAGTTGC | | |
| *Actin*_F | GAACTGGTATGGTCAAGGCTG | RT-PCR | (Chen et al. 2007) |
| *Actin*_R | ACACGGAGCTCGTTGTAGAAG | | |
| *OsRRM*_F | ACCGTCTGCTCAGGAAGACCATC | | |
| *OsRRM*_R | AAGGGAAGTGGTATTCATGGGTC | | |
| *OsRRMp*_F | GCGAGCTCTTTTTCCATCCTTGAG | Cloning *OsRRM* promoter | |
| *OsRRMp*_R | GAGGATCCCTGCAAGAACCAAGAAG | | |
| *OsRRM*_ATG | GAGGATCCATGGGGAGACCTCGAG | Cloning *OsRRM* CDS | |
| *OsRRM*_TAG | CGAAGCTTCTACTTCTGGCCACCAG | | |
| *OsRRM*-ATG | GAGTCGACATGGGGAGACCTCGAG | Cloning OsRRM (1-735 aa) | |
| *OsRRM*-RRM3' | GTCGACCTAACTGTCACTCCGAGA | | |
| *SBEIIb*-5' | GTCGACATGGCGGCGCCGGCGTCT | Cloning *SBEIIb* CDS n | |
| *SBEIIb*-3' | GAATTCTCATTCCGCTGGAGCA | | |
| *OsSUT2*-5' | GGTACCCTCTTCTGAACTAACCCAAAGAT | Cloning *OsSUT2* CDS | |
| *OsSUT2*-3' | GTCGACAACTCCTGCAACTTTTATTCACTA | | |
| *OsTMT1*-5' | GGTACCAAATCTCCCCTAAAAGCTTCC | Cloning *OsTMT1* CDS | |
| *OsTMT1*-3' | GTCGACGAACTAGTACTCGGCTATGCTAA | | |
| *OsTMT2*-5' | GGTACCAAGAGGTGGAAGAAGAGGGAT | Cloning *OsTMT2* CDS | |
| *OsTMT2*-3' | GTCGACTATTATGAACCCCAACATAGTAGC | | |

## Supporting Reference


Chen S, Wang Z, Cai X (2007) *OsRRM*, a Spen-like rice gene expressed specifically in the endosperm. **Cell Res** 17: 713-721

Cho JI, Burla B, Lee DW, Ryoo N, Hong SK, Kim HB, Eom JS, Choi SB, Cho MH, Bhoo SH, Hahn TR, Neuhaus HE, Martinoia E, Jeon JS (2010) Expression analysis and functional characterization of the monosaccharide transporters, *OsTMT*s, involving vacuolar sugar transport in rice (*Oryza sativa*). **New Phytol** 186: 657-668

Liu D, Cai X (2013) *OsRRMh*, a Spen-like gene, plays an important role during the vegetative to reproductive transition in rice. **J Integr Plant Biol** 55: 876-887

Hirose T, Zhang Z, Miyao A, Hirochika H, Ohsugi R, Terao T (2010) Disruption of a gene for rice sucrose transporter, OsSUT1, impairs pollen function but pollen maturation is unaffected. **J Exp Bot** 61: 3639-3646

Eom JS, Cho JI, Reinders A, Lee SW, Yoo Y, Tuan PQ, Choi SB, Bang G, Park YI, Cho MH, Bhoo SH, An GH, Hahn TR, Ward JM, Jeon JS (2011) Impaired function of the tonoplast-localized sucrose transporter in rice, OsSUT2, limits the transport of vacuolar reserve sucrose and affects plant growth. **Plant Physiol** 157: 109-119

Wang Y, Xiao Y, Zhang Y, Chai C, Wei G, Wei X, Xu H, Wang M, Ouwerkerk PB, Zhu Z (2008) Molecular cloning, functional characterization and expression analysis of a novel monosaccharide transporter gene *OsMST6* from rice (*Oryza sativa* L.). **Planta** 228: 525-535

Zhang H, Liang W, Yang X, Luo X, Jiang N, Ma H, Zhang D (2010) Carbon starved anther encodes a MYB domain protein that regulates sugar partitioning required for rice pollen development. **Plant Cell** 22: 672-689
